# The effects of three-dimensional defects on one-way surface plasmon propagation for photonic topological insulators comprised of continuum media

**DOI:** 10.1038/srep30055

**Published:** 2016-07-21

**Authors:** S. Ali Hassani Gangaraj, Andrei Nemilentsau, George W. Hanson

**Affiliations:** 1 Department of Electrical Engineering and Computer Science, University of Wisconsin-Milwaukee, Milwaukee, Wisconsin 53211, USA; 2 Department of Electrical and Computer Engineering, University of Minnesota, Minneapolis, Minnesota 55455, USA

## Abstract

We have investigated one-way surface plasmon-polaritons (SPPs) at the interface of a continuum magnetoplasma material and metal, in the presence of three-dimensional surface defects. Bulk electromagnetic modes of continuum materials have Chern numbers, analogous to those of photonic crystals. This can lead to the appearance of topologically-protected surface modes at material interfaces, propagating at frequencies inside the bandgap of the bulk materials. Previous studies considered two-dimensional structures; here we consider the effect of three-dimensional defects, and show that, although backward propagation/reflection cannot occur, side scattering does take place and has significant effect on the propagation of the surface mode. Several different waveguiding geometries are considered for reducing the effects of side-scattering, and we also consider the effects of metal loss.

Extensive research has been carried out in the field of surface plasmon-polaritons (SPPs) due to their technological potential and fundamental nature^1^,^2^. Recently, exciting phenomena have been studied such as unidirectional plasmon coupling^3^, plasmon focusing^4^,^5^, waveguiding and interferometry^6^,^7^ and planar optical chirality^8^,^9^. It has also been shown that SPP waves can be excited in a single direction using a circularly-polarized source that couples to SPP spin polarization^10^. However, upon encountering a discontinuity, partial reflection of the wave will occur since the material itself generally allows propagation in all directions. But, if the medium itself only supports modes that can travel in one direction, then upon encountering a discontinuity an SPP cannot be reflected (back-scattered). This is a rather remarkable occurrence, and has important applications in waveguiding applications (e.g., defect-immune waveguides).

More generally, a broad class of materials known as photonic topological insulators (PTIs) provide backscattering-immune unidirectional mode propagation^11^,^12^,^13^,^14^,^15^,^16^,^17^,^18^,^19^,^20^,^21^,^22^,^23^. The ideas underlying unidirectional transport were initially developed for electrons in crystalline materials (electronic topological insulators, Chern insulators, and quantum Hall effect (QHE) materials)^24^,^25^. In the photonic case, the most common, and historically first approach^11^,^14^ utilizes two-dimensional (2D) photonic crystals with degenerate Dirac cones in their photonic band structure. The degeneracy can be lifted by breaking time-reversal (TR) symmetry, which opens a band gap and leads to topologically non-trivial photonic bands with non-zero Chern numbers. The unidirectional SPP mode can then propagate at the interface between such a material and an ordinary photonic insulator or simple opaque material. Time-reversal symmetry can be lifted by applying a static magnetic field to a gyromagnetic material^15^,^16^, time-harmonic modulation of coupled dielectric resonators^17^,^19^, or by translating the modulation from time-domain to spatial domain^20^. Unidirectional transport of photons in this case is analogous to the unidirectional electronic edge currents in the quantum Hall effect^11^ and thus the above case can be referred to as a photonic quantum Hall effect (PQHE). Alternatively, photonic analogues of electronic topological insulators utilizing time-reversal-invariant topologically protected *Z*_2_ states were proposed^18^,^21^,^26^. In this case, photons are separated into different ‘spin’ sub-spaces based on their polarization, and “spin-orbit” coupling is introduced through exploiting such non-trivial characteristics of metamaterials as chirality, bi-anisotropy and hyperbolicity. In general, both types of structures can be classified as being PTI, since in each of the cases the material itself is a bulk electromagnetic insulator supporting conducting surface states. In this paper we focus on the material that belongs to the PQHE type.

Most PTI implementations utilize a spatially periodic medium where the underlying wave vector space is isomorphic to a torus^27^. However, the possibility of continuum (non-periodic) materials, either homogenized metamaterials or natural materials, to support topologically protected unidirectional photonic surface states has been also recently been shown^28^,^29^,^30^,^31^,^32^. The wave vector space in this case is the 2D plane mapped to the Riemann sphere. In fact, existence of the immune to backscattering edge/surface modes were initially predicted for the case of continuum medium, i.e for the interface between a biased magnetoplasma and metal^30^. The formalism that allows for the formal extension of the concept of PTI to the case of continuous media based on global features of the eigenmodes has been developed in ref. 31. In this case, the Chern number is calculated for a set of eigenmodes of the continuous material (rather than for the photonic bands). The Chern number computed in such a way has analogous meaning to that for electronic or photonic bands in a periodic structure. In particular, at the interface between two materials, the number of one-way states is equal to the difference of gap Chern numbers between the materials, where the gap Chern number 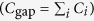 is the sum of the Chern numbers of all modes below the band gap^28^,^33^,^34^,^35^. If one of the materials is topologically trivial (*C*_gap_ = 0) like metal or air, the other gap Chern number determines the number of topologically protected one-way interface states. Alternatively, Chern numbers can be defined for equi-frequency surfaces (EFS)^28^,^29^.

Previous works on PTIs considered 2D structures and demonstrated immunity of topologically protected one-way interface modes with respect to backscattering by 2D defects. However, most realistic waveguides and defects are necessarily three-dimensional (3D). This fact may cause several problems with the practical implementation of PTIs. First of all, scattering of the interface mode at a 3D defect is not limited to backscattering. In fact, side scattering may be significant and PTIs seem not to offer any protection against this. Moreover, modes of the finite waveguides are cavity modes rather than modes of the infinite medium. Thus, the finite waveguide might have modes with frequencies in the bandgap region of bulk PTIs, which may lead to coupling of interface modes to the bulk cavity modes, and thus to significant decrease of the one-way mode propagation distance. In this work we consider these issues, taking the interface between a biased magnetoplasma and metal as our model system. The magnetoplasma was considered before in various contexts, such as sub-diffractional imaging^36^ and magnetic field induced transparency^37^. The topological protection of the interface mode in magnetoplasma was demonstrated^30^, although neither formal assessment of the topological bandstructure was made nor were the Chern numbers calculated (the work in refs 28 and 29 is highly related, but is based on EFS-related calculations).

In this paper, we analytically calculate Chern numbers for the eigenmodes supported by a biased magnetoplasma based on global dispersion behavior of the eigenmodes of the bulk structure, using the continuum material theory developed in ref. 31. (see Supplemental Information (SI) for details). We demonstrate that eigenmodes of bulk magnetoplasma indeed possess non-trivial Chern numbers, which leads to propagation of the topologically non-trivial surface modes at the interface between the magnetoplasma and metal that are immune to backscattering. We consider interaction of these modes with 3D defects and demonstrate that side-scattering on these defects significantly decreases SPP propagation lengths and thus has to be dealt with in any realistic waveguide. We also show that radiative losses due to the finite sizes of the waveguide can be important. We offer two different waveguide geometries that counters these effects and achieve lossless or low-loss propagation of surface modes immune to back-scattering on defects and waveguide inhomogeneities.

## Results

### Chern number and bulk-edge correspondence

The ideas underlying electronic topological insulators can be extended to the photonic case by utilizing the correspondence between Maxwell’s equations and Schrödinger’s equation^11^,^14^,^31^. We assume lossless dispersive materials^11^,^14^, such that the continuum material can be characterized by dimensionless real-valued parameters 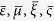, representing permittivity, permeability and magneto-electric coupling tensors. We can write Maxwell’s equations as^38^ 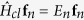, where 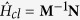 plays the role of a classical Hamiltonian,


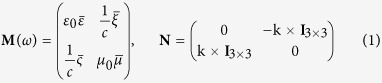


(**M** is Hermitian for (*ω*, k) real-valued), *c* is the vacuum speed of light, and **f** = [E, H]^T^. In the following we will set the magneto-electric coupling tensors to zero.

The Berry potential **A**_*n*_ = *i*〈**f**_*n*_ | ∇_**k**_**f**_*n*_〉 for the case of dispersive materials defined by **M**(*ω*), is^11^


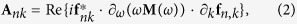


where *ω* is the radian frequency, and the Chern number can then be calculated as^31^ (see also SI for details)


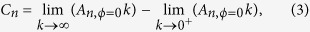


where 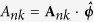.

**Figure 1:**
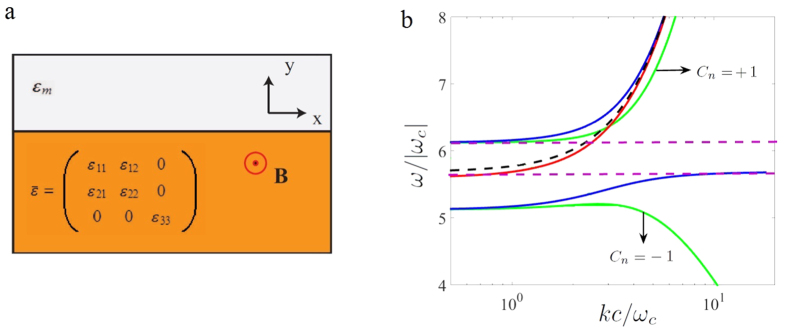
(**a**) Interface between a biased plasma (bottom) and a simple metal (top). (**b**) TM and TE band diagram for *ω*_*p*_/2*π* = 9.7 THz and *ω*_*c*_/2*π* = 1.73 THz; *ω*_*p*_ = 5.6*ω*_*c*_. Solid blue: bulk TM mode for local model, solid red: bulk TE mode for local model, solid green: bulk TM mode for nonlocal model with spacial cutoff *k*_max_ = 10|*ω*_*c*_|/*c*, dashed black: SPP mode dispersion at the interface of magnetoplasma and metal (local model). The dashed purple lines show the bulk TM mode gap (local modal).

Let us consider a magnetized plasma in the Voigt configuration and assume interface mode propagation in the direction perpendicular to the bias magnetic field **B**, as depicted in Fig. 1a. For a single-component plasma biased with a static magnetic field 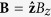, the permeability is *μ* = *μ*_0_ and the relative permittivity has the form of a Hermitian antisymmetric tensor,


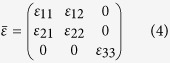


where


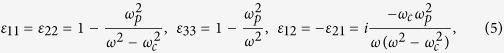


and where the cyclotron frequency is *ω*_*c*_ = (*q*_*e*_/*m*_*e*_)*B*_*z*_ and the plasma frequency is 
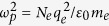
. In the above, *N*_*e*_ is the free electron density, and *q*_*e*_ and *m*_*e*_ are the electron charge and mass, respectively.

Let us first consider (bulk) photonic modes of the infinite magnetized plasma. We assume electromagnetic wave propagation in the *xoy* plane, **k** = (*k*_*x*_, *k*_*y*_, 0). In this case the transverse electric (TE) (*E*_*z*_ ≠ 0, *H*_*z*_ = 0) and transverse magnetic (TM) (*E*_*z*_ = 0, *H*_*z*_ ≠ 0) modes are decoupled and their dispersion is given by


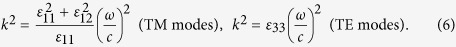


Figure 1b depicts the band diagram of the bulk TM (solid blue) and bulk TE (solid red) polarized modes for the case *ω*_*p*_ = 5.6*ω*_*c*_. The dispersion of the TE mode shows a band gap for *ω* < *ω*_*p*_ and the TM modes are organized into two branches separated by a band gap.

Using Eq. (3) (see SI for details) we calculate the Chern numbers for all bands. The Chern number for the TE band is trivial (*C*_*n*_ = 0), and the Chern number of the high-frequency TM band is 1. However, the Chern number of the low frequency TM band is not an integer, 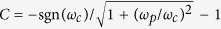, which is associated with the Hamiltonian of the medium not being sufficiently well behaved as |k| → ∞ for the local model^31^.

An integer Chern number for both branches can be obtained using a non-local material response with a high-wavenumber spatial cutoff 31, 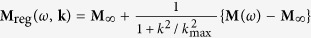, where **M**_∞_ = lim_*ω*→∞_**M**(*ω*) and the spatial cutoff *k*_max_ determines the strength of non-locality such that as *k*_max_ → ∞ the material model becomes local. For this nonlocal material response, the band diagram and Chern numbers have been obtained using *k*_max_ = 10|*ω*_*c*_|/*c* (for TM modes these are shown in Fig. 1b as solid green lines, and the Chern number calculation is detailed in the SI; the Chern number calculation is insensitive to the value of *k*_max_). As can be seen in Fig. 1, for the lower TM mode the lower frequency limit of the band gap in the presence of spacial cutoff changes somewhat from the local case (blue lines), and we have integer Chern numbers for all modes, such that *C*_gap_ = −1, which indicates the presence of one back-scattering-protected SPP. From (27) and (28) in the Supplemental information (SI), the lower and upper frequency of the TM band gap is *ω*_*L*_/*ω*_*c*_ = 5.66 and *ω*_*H*_/*ω*_*c*_ = 6.07.

Let us now consider SPP propagation at the interface between magnetized plasma and isotropic non-magnetic material. The dispersion equation of the TM-polarized SPP mode (assuming local model for dielectric response of magnetized plasma) is^30^


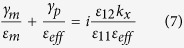


where *k*_*x*_ is the propagation constant of the interface state, *ε*_*m*_ is the permittivity of the metal, *ε*_11_ and *ε*_12_ describe the permittivity of the magnetoplasma material (5), 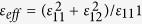, 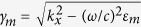 and 
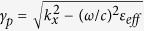
. In the following results we consider silver as the metal, which, at *ω*/2*π* = 10 THz (*ω*/*ω*_*c*_ = 5.78), has permittivity *ε* = (−4 − *i*1.69) × 10^4^. In order to focus on the important physics, we first consider lossless silver (*ε* = −4 × 10^4^; the results using this value are not too different than for a perfect-electric conductor). The dashed black lines in Fig. 1b show dispersion of the SPP (edge mode). The bandgap is indicated by the dashed purple lines (for the local model). As can be seen, the SPP dispersion line crosses the bandgap of the bulk modes, which is the frequency range of interest in the following.

### Full-wave simulation of one-way interface mode propagation

Certainly, the most celebrated property of topological materials is their potential to support propagation of backscattering-protected modes at the interface between two topologically distinct materials. Furthermore, one wants to operate in the band gap of both materials (or to have the materials electromagnetically opaque), to avoid losses due to radiation and diffraction at surface discontinuities. In this section we consider one-way propagation of the surface mode at the interface between a biased magnetoplasma and metal. All numerical simulations are performed using CST Microwave Studio.

**Figure 2:**
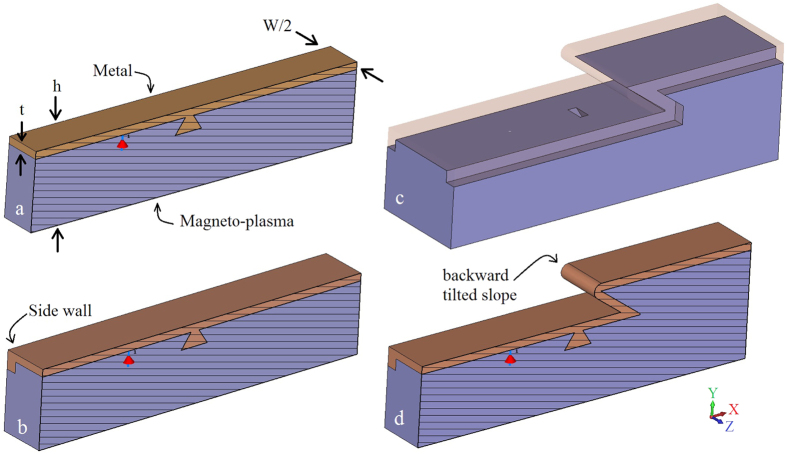
(**a**) Perspective cross section view of the interface between a metal coating (top brown material) and a magnetoplasma material (lower purple material), including a metallic pyramidal defect near a dipole source (red arrow, vertically-polarized). The entire structure has length 480 *μ*m, the width of the interface (along *z*) is *W* (either 240 *μ*m or 100 *μ*m) and the coating thickness is *t* (10 *μ*m). (**b**) Perspective cross section view of the same structure with partial side walls (which extend 15 *μ*m down the waveguide side). c- Perspective view of a plasmonic waveguide incorporating an inclined step discontinuity. d- Perspective cross section view of the waveguide with inclined step discontinuity.

Previous studies of PTIs focused on 2D waveguiding structures and considered propagation of the unidirectional modes at the interface between two semi-infinite half-spaces filled with topologically distinct materials. The 2D geometry however, can not account for the presence of 3D defects, which is a more realistic scenario. In the case of a unidirectional mode interacting with such a defect, the energy can be scattered sideways (upwards/downwards scattering/radiation into the bulk is assumed suppressed due to being in the bandgap of the bulk materials). It is obvious that the lack of backward states prohibits backward scattering of the mode. However, sideways scattering can still be an issue as it is allowed. Thus, it is important to estimate how significant this decay channel is. To address this issue, we study excitation of the TM mode at the interface between a magnetized plasma and metal for several waveguiding geometries (see Fig. 2). We chose an electrical dipole oriented normal to the interface as the excitation source (red arrow in Fig. 2), as it couples predominantly to TM modes. In what follows we assume that the dipole frequency is in the band gap of the magnetoplasma material, *ω*/2*π* = 10 THz (*ω*/*ω*_*c*_ = 5.78 THz).

The first waveguiding geometry (cross-section is shown in Fig. 2a) consists of a thin metal layer (thickness *t*, width *W*) placed on top of a magnetoplasma material (thickness *h* − *t*, width *W*). We added a metallic pyramid at the interface to serve as a 3D defect. The size of this defect is comparable to the SPP wavelength, such that the path along the contour of the defect is approximately a half wavelength of the SPP (*λ*_SPP_ ≈ 162 *μ*m). The waveguide is 480 *μ*m long (x-direction, ≈3*λ*_SPP_), the right end is at *x* = 350 *μ*m, and the left end is at *x* = −130 *μ*m); the source is at *x* = 0 centered across the width of the structure (*z* = 0). The ends are terminated by an open circuit (vacuum). In the following, we consider two widths (along *z*), a wide waveguide, *W* = 240 *μ*m (1.48*λ*_SPP_) and a narrower waveguide, *W* = 100 *μ*m (0.62*λ*_SPP_). The coating thickness is *t* = 10 *μ*m (0.06*λ*_SPP_), the depth is *h* = 95 *μ*m (0.59*λ*_SPP_), and we assume lossless or lossy silver as the metal. For the structure with partial sidewalls, the extension of the side wall down the sides of the waveguide is 15 *μ*m (0.09*λ*_SPP_).

**Figure 3:**
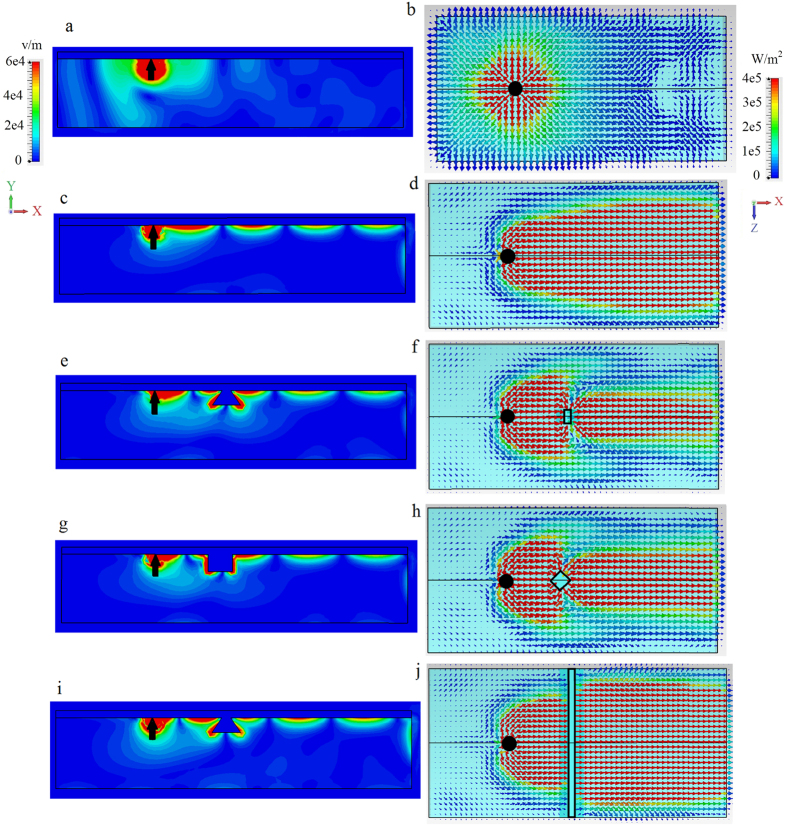
Wide waveguide, no sidewalls. Electric field and power distribution for the surface mode excited by electric dipole (black arrow and circle) at the interface between metal and magnetoplasma (*ω*_*p*_/2*π* = 9.7 THz), as shown in Fig. 2a, for a wide waveguide, *W* = 240 *μ*m (1.48*λ*_SPP_). Left panels: distribution of the electric field intensity in the *x* − *y* plane at the cross-section *z* = 0. Right panels: Top view of power distribution at the interface. (**a,b**) Non-biased (*ω*_*c*_ = 0) magnetoplasma, no defect. (**c,d**). Biased (*ω*_*c*_/2*π* = 1.73 THz) magnetoplasma, no defect. (**e,f**). Biased magnetoplasma with 3D scatterer/defect, (**g,h**). Same as in panels (**e,f**) but for a different (diamond-shaped) 3D defect. (**i,j**). Same as in panels (**e,f**) but for a quasi-2D rectangular defect that spans the width of the waveguide.

First, we consider excitation of the surface wave at the interface between lossless silver and non-biased magnetoplasma (*ω*_*c*_ = 0) for the wide waveguide, *W* = 240 *μ*m (1.48*λ*_SPP_), in order to minimize the effects of the finite-width of the structure and to illustrate the basic physics. The distributions of the electric field and power carried by the wave for the unbiased case are presented in Fig. 3a,b, respectively. For this case time reversal symmetry is preserved and so the excitation propagates in all directions. The mode is not well-confined to the interface, which is easy to verify by obtaining the decay constant from (7) for the non-biased case (*ε*_12_ = *ε*_21_ = 0), 
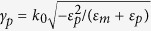
, where *ε*_11_ = *ε*_22_ = *ε*_33_ = *ε*_*p*_. Next, we break TR by applying a magnetic bias, *ω*_*c*_/2*π* = 1.73 THz. Lifting time-reversal symmetry leads to appearance of topologically non-trivial photonic modes in the magnetoplasma (see Fig. 1) and thus to the presence of unidirectional surface modes at the interface (Fig. 3c,d). One can clearly see unidirectional propagation of the mode from the power distribution presented in Fig. 3d.

As the waveguide is finite in the *z* direction, power carried in that direction (due to the omnidirectional 3D source) is radiated into free-space at the waveguide edges, leading to radiative damping of the surface wave. The radiative losses due to the excitation by the dipole can however be eliminated by choosing a source that excites only modes propagating along the *x* direction (a planar Yagi-Uda or waveguide source, for example). However, as shown in Fig. 3e,f scattering by a 3D defect also results in energy propagating transverse to the direction of propagation, and thus has to be addressed in a different way. Figure 3g,h show the effect of a different 3D defect. In both cases the 3D defect clearly has a strong impact on energy flow compared to the defect-less case, Fig. 3c,d. Finally, Fig. 3i,j shows a rectangular defect that spans the width of the waveguide, acting as a quasi-2D defect. In this case, although the defect is larger than the others, energy is able to pass through the defect without side-scattering; Fig. 3j resembles the defect-free case Fig. 3d.

**Figure 4:**
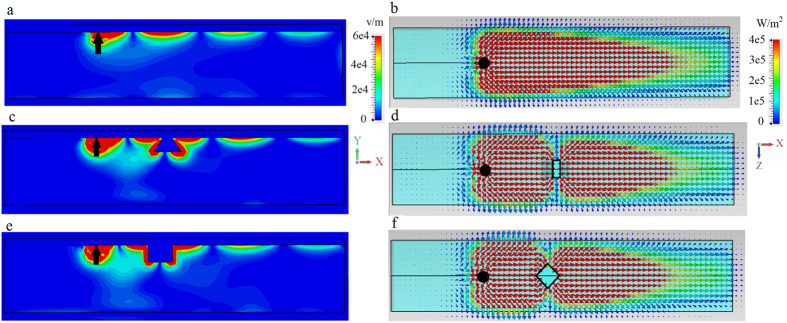
Narrow waveguide, no sidewalls. Electric field and power distribution for the surface mode excited by electric dipole (black arrow and circle) at the interface between metal and biased magnetoplasma (*ω*_*p*_/2*π* = 9.7 THz, *ω*_*c*_/2*π* = 1.73 THz), as shown in Fig. 2a, for the narrower waveguide, *W* = 100 *μ*m (0.62*λ*_SPP_). Left panels: distribution of the electric field intensity in the *x* − *y* plane at the cross-section *z* = 0. Right panels: Top view of power distribution at the interface. (**a,b**) Biased (*ω*_*c*_/2*π* = 1.73 THz) magnetoplasma, no defect. (**c,d**). Biased magnetoplasma with 3D defect. (**e,f**). Same as (**c,d**) but for a different (diamond-shaped) 3D defect.

**Figure 5:**
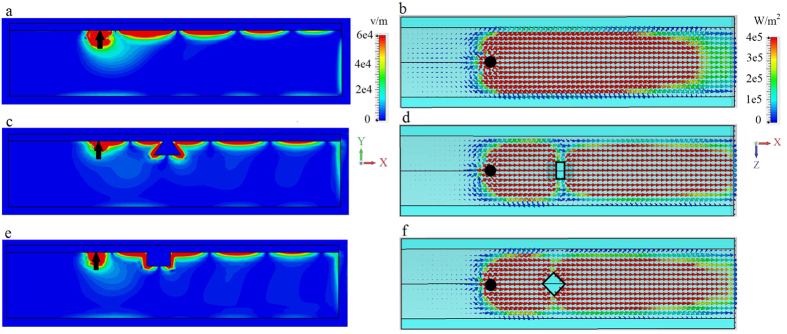
Narrow waveguide, partial sidewalls. Electric field and power distribution for the surface mode excited by electric dipole (black arrow and circle) at the interface between metal and biased magnetoplasma (*ω*_*p*_/2*π* = 9.7 THz, *ω*_*c*_/2*π* = 1.73 THz), *W* = 100 *μ*m (0.62*λ*_SPP_), with partial sidewalls (see Fig. 2b) extending 0.09*λ*_SPP_ down the waveguide sides. Left panels: distribution of the electric field intensity in the *x* − *y* plane at the cross-section *z* = 0. Right panels: Top view of power distribution at the interface. (**a,b**) Biased (*ω*_*c*_/2*π* = 1.73 THz) magnetoplasma, no defect. (**c,d**). Biased magnetoplasma with 3D defect. (**e,f**). Same as (**c,d**) but for a different (diamond-shaped) 3D defect.

The wide waveguide considered in Fig. 3 demonstrates the basic physics, but is likely too wide to be practical. In Fig. 4 we consider a narrower waveguide, *W* = 100 *μ*m (0.62*λ*_SPP_). We omit the unbiased case, and Fig. 4a–f is the same as Fig. 3c–h except for the narrower waveguide width. It is clear that the energy scattered from the defect significantly interacts with the side of the waveguide, producing radiation and commensurate increased surface wave decay/decreased propagation length. In both Figs 3 and 4, radiation into the space outside the waveguide occurs, more significantly for the narrower waveguide. In order to efficiently channel power along the propagation direction, we extended the metal cover layer partially down the sides of the waveguide; see Fig. 2b. These partial side walls are effective since the surface wave is confined near the interface (and so they need not extend down the length of the side), and act to reflect energy traveling towards the waveguide side that would otherwise be radiated. Figure 5 shows that this leads to improved confinement of the surface wave in the waveguide, decreased radiative losses due to wave components propagating in the transverse (*z*) direction, and thus to the increase of the surface polariton propagation distance.

Nevertheless, for the narrow waveguide we can still observe that the surface plasmon experiences damping. With transverse radiation mostly eliminated by the partial sidewalls, this is due to coupling to bulk modes of the finite structure. That is, the indicated bulk bandgap is for infinite materials, and, since the waveguide is finite both in *y* and *z* directions (see Fig. 2) the waveguide eigenmodes are cavity modes rather than bulk modes. Thus, a few of the cavity modes can occur in the bulk bandgap and the surface plasmon can couple to these modes, leaking energy through the bottom of the waveguide and the portion of the waveguide sides not covered by metal. This is supported by examination of Fig. 5a,c,e, where electric field penetration inside the magnetoplasma can be clearly seen.

**Figure 6:**
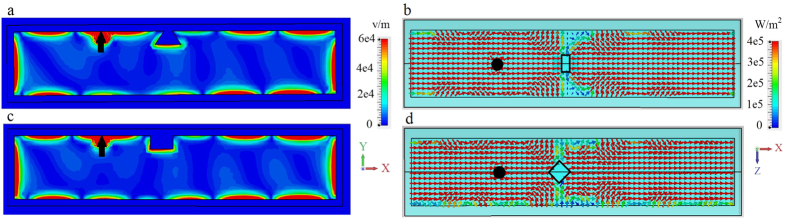
Narrow waveguide, enclosed by metal. Electric field and power distribution for the surface mode excited by electric dipole (black arrow and circle) at the interface between metal and biased magnetoplasma (*ω*_*p*_/2*π* = 9.7 THz, *ω*_*c*_/2*π* = 1.73 THz), *W* = 100 *μ*m (0.62*λ*_SPP_), fully enclosed by metal. Left panels: distribution of the electric field intensity in the *x* − *y* plane at the cross-section *z* = 0. Right panels: Top view of power distribution at the interface. (**a,b**) Biased (*ω*_*c*_/2*π* = 1.73 THz) magnetoplasma with 3D defect. (**c,d**). Same as (**a,b**) but for a different (diamond-shaped) 3D defect.

In general, power loss can be overcome by enclosing all of the faces of the waveguide by a metal layer (here, chosen to be 10 *μ*m thick), as shown in Fig. 6. The plasmon circulates the entire structure in a clockwise manner, without any losses. There is, in fact, coupling of energy to the bulk modes, but this is eventually returned back to the surface mode (some energy also circulates in the *y* − *z* plane, around the circumference of the structure, due to side scattering by the defect). By opening the end-faces of the structure (planes perpendicular to *x* direction) we can let energy out, thus implementing a unidirectional waveguide without losses. Reversing the direction of the magnetic field results in counter-clockwise rotation of the plasmon. Videos showing clockwise and counterclockwise rotation of the SPP for positive and negative bias, respectively, are available in the Supplemental Information.

**Figure 7:**
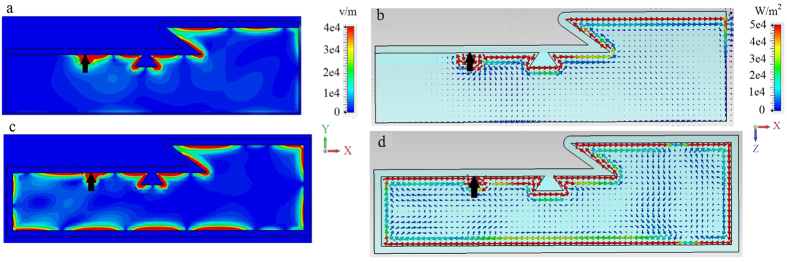
Narrow waveguide, defect and inclined slope. Electric field and power distribution for the surface mode excited by electric dipole (black arrow and circle) at the interface between metal and biased magnetoplasma (*ω*_*p*_/2*π* = 9.7 THz, *ω*_*c*_/2*π* = 1.73 THz), *W* = 100 *μ*m (0.62*λ*_SPP_). (**a,b**) the waveguide sides are partly covered by metal (see Fig. 2c,d), (**c,d**) the waveguide is enclosed by metal. Left panels: distribution of the electric field intensity in the *x* − *y* plane at the cross-section *z* = 0. Right panels: Corresponding power distribution.

As further demonstration of the uni-directional nature of this waveguiding structure, Fig. 7 shows results for a waveguide with both a defect and an inclined step discontinuity, as depicted in Fig. 2c,d. Partial side walls are again used to partially prevent field leakage into space, and the length of the tilted slope is comparable to the SPP wavelength. Figure 7 shows a side view of the electric field (left) and side view of the power distribution (right) for biased (a,b) magnetoplasma with lossless silver, and for biased magnetoplasma with a lossless silver layer surrounding all faces (c,d). Since the inclined slope defect spans the entire width of the structure, it acts as a quasi-2D defect, similar to Fig. 3i,j, and does not impede energy flow. For Fig. 7c,d, as with Fig. 6, we observe clockwise circulation of the surface wave around the structure, without any damping.

**Figure 8:**
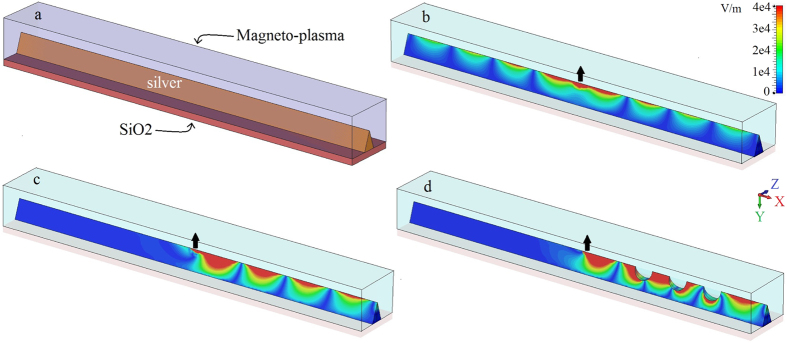
(**a**) Perspective view of the silver ridge waveguide with height 30 *μ*m and opening angle 20 degree mounted on SiO_2_ substrate and covered by magnetoplasma material. (**b**) Electric field distribution on the ridge for non-biased (*ω*_*c*_ = 0) case and (**c**) for biased (*ω*_*c*_/2*π* = 1.73 THz) case. (**d**) shows the biased case with defects on the ridge. The dipole source is indicated by a black arrow, lossy silver is considered, and in all cases the excitation frequency *ω*/2*π* = 10 THz. *ω*_*p*_/2*π* = 9.7 THz.

As a possible alternative waveguide that eliminates side-scattering, we consider a ridge waveguide, depicted in Fig. 8a. Ridge waveguides were shown to provide efficient channeling of SPPs in reciprocal photonic integrated circuits^2^,^39^,^40^,^41^,^42^. Here we study potentiality of ridge waveguides for non-reciprocal SPP channeling. The ridge is made of lossy silver with height 30 *μ*m and opening angle 20 degree placed on the top of a substrate made of SiO_2_ and covered by magnetoplasma material. The magnetoplasma material is biased along the *z* direction. A y-polarized dipole source is placed at the top of the ridge and radiates at 10 THz. Figure 8b shows the absolute value of the electric field on the ridge covered by non-biased (*ω*_*c*_/2*π* = 0) magnetoplasma. This is a reciprocal case and SPP propagates both to the left and right from dipole. If we turn the bias on (*ω*_*c*_/2*π* = 1.73 THz), as shown in Fig. 8c, we switch to the non-reciprocal regime and get SPP propagating only toward right without any backward propagation. Although it is not shown here, the direction of SPP propagation can be reversed by reversing the direction of magnetic bias (i.e. from right to left by changing bias direction from *z* to −*z*). Figure 8d shows the one-way propagation of the SPP in the presence of defects in the ridge waveguide (defects are regions removed from the silver material (i.e., ‘holes’)). As expected, the defect does not scatter energy, and the SPP propagates around the defect. One could also consider a circular cross-section geometry (e.g., a magnetoplasma circular cross-section cylinder surrounded by metal), although the proposed structures, rectangular cross-section and ridge waveguides, are more amenable to device integration.

All the analysis assumed the Voight configuration, where propagation is perpendicular to the biasing field. Existence of such a mode is valid for the case of in-plane bends. If we have a bend in the plane of the biasing field, then the propagation in the bend region is not normal to the biasing field (unless the bias direction changes with the bend). For propagation perpendicular to the bias, modes are decoupled TE and TM, whereas for propagation parallel to the bias, modes are right- and left-handed circularly polarized, which will lead to back scattering in the bend region.

Concluding, in this paper we studied the effects of 3D defects and the finite size of realistic waveguides on propagation of topologically protected unidirectional surface modes. We calculated analytically the Chern number of the bulk electromagnetic modes in a continuum biased magnetoplasma, and demonstrated the topologically non-trivial character of these modes, which leads to appearance of unidirectional surface waves at the interface between the magnetoplasma and plasmonic metal. While it is well known that suppressed backscattering allows these modes to carry energy around 2D defects in the plane of the interface without any losses, we demonstrated that 3D defects can have a substantial detrimental effect on unidirectional surface mode propagation length, due to the fact that 3D defects can scatter energy sideways. While uni-directionality prevents surface mode energy from flowing backwards, no protection is provided for energy flowing sideways. The energy leaking into free space from the sides of waveguides can lead to significant radiation losses. Another mechanism of losses is coupling of surface modes to the bulk cavity modes of realistic finite-size waveguides. Even though bulk modes in magnetoplasma have a bandgap at the frequency of waveguide operation, cavity modes can exist in the band gap, which is an inevitable consequence of the finiteness of any realistic waveguide structure. We suggested two different ways to solve the problems of side scattering and cavity modes. One consists of enclosing the magnetoplasma material with metal, partially or fully, while another relies on using a ridge waveguide.

## Additional Information

**How to cite this article**: Hassani Gangaraj, S. A. *et al*. The effects of three-dimensional defects on one-way surface plasmon propagation for photonic topological insulators comprised of continuum media. *Sci. Rep.* **6**, 30055; doi: 10.1038/srep30055 (2016).

## Supplementary Material

Supplementary Information

Supplementary Video S1

Supplementary Video S2
