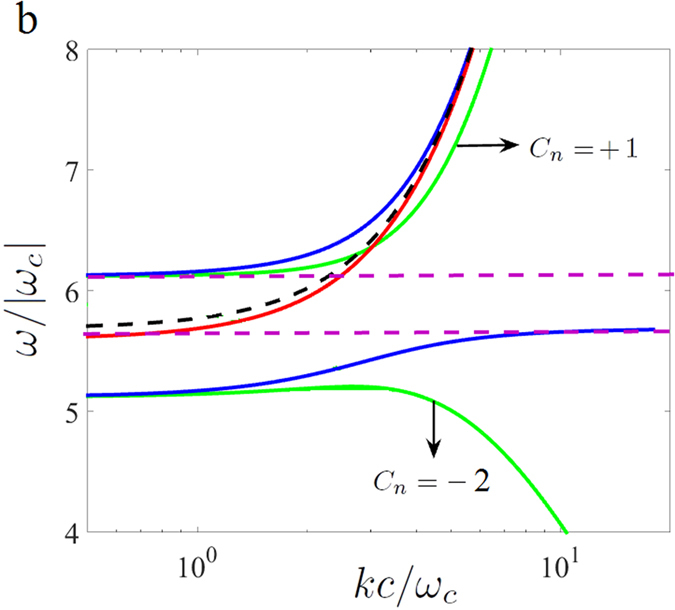# Corrigendum: The effects of three-dimensional defects on one-way surface plasmon propagation for photonic topological insulators comprised of continuum media

**DOI:** 10.1038/srep46859

**Published:** 2017-07-10

**Authors:** S. Ali Hassani Gangaraj, Andrei Nemilentsau, George W. Hanson

Scientific Reports 6: Article number: 30055; 10.1038/srep30055 published online: 07 21 2016; updated: 07 10 2017.

This Article contains errors. In the Results section, Equation (7),

“
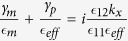
”

should read:

“
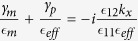
”

In the Supplementary Information, Equation (40),

“
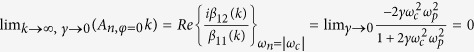
”

should read:

“
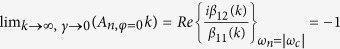
”

Additionally in the Supplementary Information, Equation (42),

“
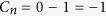
”

should read:

“
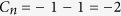
”

Note that the sum of the Chern numbers appears to be −1. As detailed in supplementary reference [1], to predict edge states in general for continuum media, one should compute Chern numbers for an “interpolated material response”. This means, for example, that to see bulk-edge correspondence for the magnetized plasma and a Drude metal interface, we should define a function 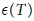 where varies from 0 to 1, such that when 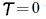 we obtain the permittivity of the magnetized plasma, and when 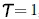, we obtain the Drude metal. Then, one needs to compute the topological numbers for 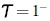 and 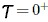. With this model, we obtain one additional low frequency band for the magnetized plasma, very near Ω = 0, having Chern number +1 which means the gap Chern number is still −1 (as indicated in page 3, main text).

Furthermore, the label “*C*_*n*_ = −2” in Figure 1b is incorrectly given as “*C*_*n*_ = −1”. The correct Figure 1b appears below as Figure 1.

## Figures and Tables

**Figure 1:**