# Supplementary material for: The effects of three-dimensional defects on one-way surface plasmon propagation for photonic topological insulators comprised of continuum media
Source: Sci Rep. 2016 Jul 21;6:30055. doi: 10.1038/srep30055 (PMC4956765; doi:10.1038/srep30055)
Supplement: Supplementary Information [file srep30055-s1.pdf]

Supplemental material for
The effects of three-dimensional defects on one-way surface plasmon propagation for photonic topological insulators comprised of continuum media

S. Ali Hassani Gangaraj, Andrei Nemilentsau, George W. Hanson

Department of Electrical Engineering, University of Wisconsin-Milwaukee
Milwaukee Wisconsin 53211, USA

Email: Hassani4@uwm.edu

June 3, 2016

1 Maxwell's equation-Schrödinger equation duality

We first assume lossless and dispersionless materials characterized by dimensionless real-valued parameter $\overline{\epsilon}$, $\overline{\mu}$, $\overline{\xi}$, $\overline{\varsigma}$, representing permittivity, permeability and magneto-electric coupling tensors. In such a medium the Maxwell's equations (considering time convention $e^{-i\omega t}$) are

$$\begin{aligned} \nabla \times \mathbf{E} &= -\mu_0 \overline{\mu} \cdot \frac{\partial \mathbf{H}}{\partial t} - \frac{\overline{\varsigma}}{c} \cdot \frac{\partial \mathbf{E}}{\partial t} - \mathbf{J}_m \\ \nabla \times \mathbf{H} &= \epsilon_0 \overline{\epsilon} \cdot \frac{\partial \mathbf{E}}{\partial t} + \frac{\overline{\xi}}{c} \cdot \frac{\partial \mathbf{H}}{\partial t} + \mathbf{J}_e. \end{aligned} \tag{1}$$

By defining the matrices

$$\boldsymbol{M} = \begin{pmatrix} \epsilon_0 \overline{\epsilon} & \frac{1}{c} \overline{\xi} \\ \frac{1}{c} \overline{\varsigma} & \mu_0 \overline{\mu} \end{pmatrix}, \ \hat{N} = \begin{pmatrix} 0 & i \nabla \times \mathbf{I}_{3\times3} \\ -i \nabla \times \mathbf{I}_{3\times3} & 0 \end{pmatrix}, \tag{2}$$

$$\boldsymbol{f} = \begin{pmatrix} \mathbf{E} \\ \mathbf{H} \end{pmatrix}, \ \boldsymbol{g} = \begin{pmatrix} \mathbf{D} \\ \mathbf{B} \end{pmatrix} = \boldsymbol{M} \cdot \boldsymbol{f}, \ \boldsymbol{J} = \begin{pmatrix} \mathbf{J}_e \\ \mathbf{J}_m \end{pmatrix}$$

where $\boldsymbol{M}$ is Hermitian and real-valued, we can write Maxwell's equations in a compact form [1],

$$\hat{N} \cdot \boldsymbol{f} = i \left[\frac{\partial \boldsymbol{g}}{\partial t} + \boldsymbol{J} \right] = i \left[\boldsymbol{M} \frac{\partial \boldsymbol{f}}{\partial t} + \frac{\partial \boldsymbol{M}}{\partial t} \boldsymbol{f} + \boldsymbol{J} \right]. \tag{3}$$

Note that the units of the sub-blocks of $\boldsymbol{M}$ differ (as do the dimensions of the 6-vectors $\boldsymbol{f}$ and $\boldsymbol{g}$) , and that ϵ, ξ, ς, and μ are dimensionless. In the absence of an external excitation ($\boldsymbol{J} = 0$) and assumption of non-dispersive (instantaneous) materials, Maxwell's equations become

$$i \frac{\partial \boldsymbol{f}}{\partial t} = \hat{H}_{cl} \cdot \boldsymbol{f} \tag{4}$$

where $\hat{H}_{cl} = \boldsymbol{M}^{-1} \cdot \hat{N}$, which has the same form as the Schrödinger equation with $\hbar = 1$, where the operator H_{cl} plays the role of a classical Hamiltonian. Because of this similarity between Maxwell's equations and the Schrödinger equation it is straightforward to extend the Berry potential concept to electromagnetic energy (photons); rather then, say, electrons acquiring a Berry phase while transversing a path in parameters space,

photons will do the same (the polarization of the photon plays the role of particle spin). In this case, we define $\boldsymbol{f}_n$ as a six-component eigenmode satisfying

$$\hat{H}_{cl} \cdot \boldsymbol{f}_n = E_n \boldsymbol{f}_n \tag{5}$$

where $E_n = \omega_n$. Equation (5) applies also to lossless dispersive materials, which we consider in the following. Assuming the normalization condition $\langle f_n | f_m \rangle = \delta_{nm}$, the Berry vector potential is

$$\mathbf{A}_n = i \langle \boldsymbol{f}_n | \nabla_{\mathbf{k}} \boldsymbol{f}_n \rangle \,. \tag{6}$$

such that $\nabla_{\mathbf{k}}$ operates over parameter space $\mathbf{k} = (k_x, \ k_y, \ k_z)$. In [2], it was demonstrated by Raghu and Haldane that this result can be extended even for dispersive materials, $\boldsymbol{M}(\omega)$, and the Berry vector potential takes the following form

$$\boldsymbol{A}_{nk} = Re \left\{ \frac{1}{2} i \boldsymbol{f}^*_{nk} \cdot \left[\frac{\partial(\omega \boldsymbol{M})}{\partial \omega} \right]_{\omega_{n,k}} \cdot \partial_k \boldsymbol{f}_{nk} \right\} \tag{7}$$

with the inner product

$$\langle \boldsymbol{f}_n | \boldsymbol{f}_m \rangle = \frac{1}{2} \boldsymbol{f}^*_n \frac{\partial \left(\omega \boldsymbol{M} \left(\omega \right) \right)}{\partial \omega} \boldsymbol{f}_m \tag{8}$$

We consider a magnetized plasma in the Voigt configuration (propagation perpendicular to the bias magnetic field $\mathbf{B}$), as depicted in Fig. 1 in the main text.

2 Magnetically biased plasma continuum model

For a single-component plasma biased with a static magnetic field $\mathbf{B} = \mathbf{z} B_z$, the permeability is $\mu = \mu_0$ and the relative permittivity has the form of a Hermitian antisymmetric tensor,

$$\bar{\epsilon} = \begin{pmatrix} \epsilon_{11} & \epsilon_{12} & 0 \\ \epsilon_{21} & \epsilon_{22} & 0 \\ 0 & 0 & \epsilon_{33} \end{pmatrix} \tag{9}$$

where

$$\begin{aligned} \varepsilon_{11} = \varepsilon_{22} &= 1 - \frac{\omega_p^2}{\omega^2 - \omega_c^2}, \quad \varepsilon_{33} = 1 - \frac{\omega_p^2}{\omega^2}, \\ \varepsilon_{12} = -\varepsilon_{21} &= i \frac{-\omega_c \omega_p^2}{\omega \left(\omega^2 - \omega_c^2 \right)} \end{aligned} \tag{10}$$

where the cyclotron frequency is $\omega_c = (q_e/m_e) \, B_z$ and the plasma frequency is $\omega_p^2 = N_e q_e^2 / \varepsilon_0 m_e$. In the above, N_e is the free electron density, and q_e and m_e are the electron charge and mass, respectivly.

The associated electromagnetic waves envelopes can be obtained by finding the solution $f = [\mathbf{E}, \mathbf{H}]^T$, of (5), $N \cdot f = \omega M \cdot f$, which is

$$\begin{pmatrix} 0 & -\mathbf{k} \times \mathbf{I}_{3\times3} \\ \mathbf{k} \times \mathbf{I}_{3\times3} & 0 \end{pmatrix} \cdot \begin{pmatrix} \mathbf{E} \\ \mathbf{H} \end{pmatrix} = \begin{pmatrix} \omega \epsilon_0 \bar{\epsilon} & 0 \\ 0 & \omega \mu_0 \mathbf{I}_{3\times3} \end{pmatrix} \cdot \begin{pmatrix} \mathbf{E} \\ \mathbf{H} \end{pmatrix} \tag{11}$$

so that

$$\begin{pmatrix} -\mathbf{I}_{3\times3} & -\frac{\bar{\epsilon}^{-1}}{\omega\epsilon_0} \cdot \mathbf{k} \times \mathbf{I}_{3\times3} \\ \frac{1}{\omega\mu_0} \cdot \mathbf{k} \times \mathbf{I}_{3\times3} & -\mathbf{I}_{3\times3} \end{pmatrix} \cdot \begin{pmatrix} \mathbf{E} \\ \mathbf{H} \end{pmatrix} = 0. \tag{12}$$

With $\mathbf{H} = \widehat{\mathbf{z}} \rightarrow \mathbf{E} = \bar{\epsilon}^{-1} \cdot \frac{\widehat{\mathbf{z}} \times \mathbf{k}}{\omega \epsilon_0}$ (TM), $\mathbf{E} = \widehat{\mathbf{z}} \rightarrow \mathbf{H} = \frac{\mathbf{k}}{\omega \mu_0} \times \widehat{\mathbf{z}}$ (TE), we have the 6×1 vectors

$$\begin{aligned} \boldsymbol{f}^{\mathrm{TM}}_{nk} &= \begin{pmatrix} \bar{\epsilon}^{-1} \cdot \widehat{\mathbf{z}} \times \frac{\mathbf{k}}{\epsilon_0 \omega_{nk}} \\ \widehat{\mathbf{z}} \end{pmatrix}, \\ \boldsymbol{f}^{\mathrm{TE}}_{nk} &= \begin{pmatrix} \widehat{\mathbf{z}} \\ \frac{\mathbf{k}}{\mu_0 \omega_{nk}} \times \widehat{\mathbf{z}} \end{pmatrix}. \end{aligned} \tag{13}$$

Because the envelopes of the electromagnetic waves in the above equations are not normalized, the Berry potential is computed using

$$\mathbf{A}_{nk} = \frac{\mathrm{Re}\{i\boldsymbol{f}^*_{nk} \cdot \frac{\partial}{\partial\omega}(\omega\boldsymbol{M}(\omega)) \cdot \partial_k \boldsymbol{f}_{n,k}\}}{\boldsymbol{f}^*_{nk} \cdot \frac{\partial}{\partial\omega}(\omega\boldsymbol{M}(\omega)) \cdot \boldsymbol{f}_{n,k}}. \tag{14}$$

Considering the Riemann sphere mapping of the $k_x - k_y$ plane as detailed in [3], it is possible to write the Chern number associated with nth eigenmode branch as

$$C_n = \frac{1}{2\pi}\int \mathbf{A}_{n,k=\infty} \cdot d\mathbf{l} - \frac{1}{2\pi}\int \mathbf{A}_{n,k=0^+} \cdot d\mathbf{l} \tag{15}$$

where the two line integrals are over infinite and infinitesimal radii (north and south poles of the Riemann sphere), respectively. If we define $A_{nk} = \mathbf{A}_{nk} \cdot \hat{\phi}$ then we have

$$C_n = \lim_{k\to\infty}(A_{n,\phi=0}k) - \lim_{k\to 0^+}(A_{n,\phi=0}k). \tag{16}$$

For a lossless TM-mode in propagating in the $x-y$ plane we have $k = k_x\hat{\mathbf{x}} + k_y\hat{\mathbf{y}} = k\cos(\phi)\hat{\mathbf{x}} + k\sin(\phi)\hat{\mathbf{y}}$. Writing

$$\bar{\epsilon}^{-1} = \begin{pmatrix} \alpha_{11} & \alpha_{12} & 0 \\ \alpha_{21} & \alpha_{22} & 0 \\ 0 & 0 & \alpha_{33} \end{pmatrix} \tag{17}$$

we have

$$f_{nk} = \begin{pmatrix} \bar{\epsilon}^{-1}\cdot\widehat{\mathbf{z}} \times \frac{\mathbf{k}}{\epsilon_0\omega_{nk}} \\ \widehat{\mathbf{z}} \end{pmatrix} = \begin{pmatrix} \frac{-\alpha_{11}k_y + \alpha_{12}k_x}{\epsilon_0\omega_n} \\ \frac{-\alpha_{21}k_y+\alpha_{22}k_x}{\epsilon_0\omega_n} \\ 0 \\ 0 \\ 0 \\ 1 \end{pmatrix}, \quad \partial_k f_{nk} = \begin{pmatrix} \frac{-\alpha_{11}\hat{y}+\alpha_{12}\hat{x}}{\epsilon_0\omega_n} \\ \frac{-\alpha_{21}\hat{y}+\alpha_{22}\hat{x}}{\epsilon_0\omega_n} \\ 0 \\ 0 \\ 0 \\ 0 \end{pmatrix} \tag{18}$$

where

$$\alpha_{11} = \frac{\epsilon_{22}}{\epsilon_{11}\epsilon_{22} - \epsilon_{12}\epsilon_{21}}, \quad \alpha_{22} = \frac{\epsilon_{11}}{\epsilon_{11}\epsilon_{22} - \epsilon_{12}\epsilon_{21}}, \quad \alpha_{12} = \frac{-\epsilon_{12}}{\epsilon_{11}\epsilon_{22} - \epsilon_{12}\epsilon_{21}}, \quad \alpha_{21} = \frac{-\epsilon_{21}}{\epsilon_{11}\epsilon_{22} - \epsilon_{12}\epsilon_{21}}, \tag{19}$$

such that

$$f^*_{nk} = \frac{1}{\epsilon_0\omega_n}\begin{pmatrix} (-\alpha_{11}k_y + \alpha_{12}k_x)^* & (-\alpha_{21}k_y + \alpha_{22}k_x)^* & 0 & 0 & 0 & 1 \end{pmatrix}. \tag{20}$$

From the frequency derivative of the material response matrix, $\partial_\omega(\omega M)$, we have $\beta_{ij} = \partial_\omega(\omega\epsilon_0\epsilon_{ij})$. So, for the Berry potential we have

$$\mathbf{A}_{nk} = \frac{\mathrm{Re}\{i\boldsymbol{f}^*_{nk} \cdot \frac{\partial}{\partial\omega}(\omega\boldsymbol{M}(\omega)) \cdot \partial_k \boldsymbol{f}_{n,k}\}}{\boldsymbol{f}^*_{nk} \cdot \frac{\partial}{\partial\omega}(\omega\boldsymbol{M}(\omega)) \cdot \boldsymbol{f}_{n,k}} = \frac{Re\{N_x + N_y\}}{D} \tag{21}$$

where

$$N_x = \frac{i}{2(\epsilon_0\omega_n)^2}\{-2\alpha_{11}\alpha_{12}[k_x\beta_{12} + k_y\beta_{11}] + (|\alpha_{11}|^2 + |\alpha_{12}|^2)[k_x\beta_{11} - k_y\beta_{12}]\}\hat{x} \tag{22}$$

$$N_y = \frac{i}{2(\epsilon_0\omega_n)^2}\{2\alpha_{11}\alpha_{12}[k_x\beta_{11} - k_y\beta_{12}] + (|\alpha_{11}|^2 + |\alpha_{12}|^2)[k_x\beta_{12} + k_y\beta_{11}]\}\hat{y}$$

$$D = \frac{|k|^2}{2(\epsilon_0\omega_n)^2}[(|\alpha_{11}|^2 + |\alpha_{12}|^2)\beta_{11} - 2\alpha_{11}\alpha_{12}\beta_{12}] + \mu_0. \tag{23}$$

Therefore, for the Chern number calculation we obtain

$$A_n = \mathbf{A}_n \cdot \hat{\phi} = \frac{Re\{N_y cos(\phi) - N_x sin(\phi)\}}{D} \tag{24}$$

$$A_n(\phi = 0) = \frac{Re\{N_y(\phi = 0)\}}{D}, \quad N_y(\phi = 0) = \frac{ik}{(\epsilon_0\omega_n)^2}\{2\alpha_{11}\alpha_{12}\beta_{11} + (|\alpha_{11}|^2 + |\alpha_{12}|^2)\beta_{12}\}$$

$$A_n(\phi = 0)k = \frac{Re(\frac{i|k|^2}{(\epsilon_0\omega_n)^2}\{2\alpha_{11}\alpha_{12}\beta_{11} + (|\alpha_{11}|^2 + |\alpha_{12}|^2)\beta_{12}\})}{\frac{|k|^2}{(\epsilon_0\omega_n)^2}[(|\alpha_{11}|^2 + |\alpha_{12}|^2)\beta_{11} - 2\alpha_{11}\alpha_{12}\beta_{12}] + \mu_0}. \tag{25}$$

These expressions are used below in calculating the Chern number from (16).

Regarding to these parameters defined in previous section, the dispersion of the the TM mode is as follow

$$k^2 = \frac{\epsilon_{11}^2 + \epsilon_{12}^2}{\epsilon_{11}}(\frac{\omega}{c})^2$$
$$k^2 = \frac{\omega^2(\omega^2 - \omega_c^2) - 2\omega^2\omega_p^2 + \omega_p^4}{\omega^2 - \omega_c^2 - \omega_p^2}\frac{1}{c^2} \tag{26}$$

For the TM mode band as $k = \frac{\omega^2(\omega^2-\omega_c^2)-2\omega^2\omega_p^2+\omega_p^4}{\omega^2-\omega_c^2-\omega_p^2}\frac{1}{c^2} \to \infty$ we get the eigen frequency $\omega_n \to \infty$ or the following eigen frequency

$$\omega^2 - \omega_c^2 - \omega_p^2 = 0 \to \omega_n = \sqrt{\omega_c^2 + \omega_p^2} \tag{27}$$

such that $\omega_n \to \infty$ belongs to the high frequency band and $\omega_n = \sqrt{\omega_c^2 + \omega_p^2}$ belongs to the low frequency band.

For the TM mode if $k = \frac{\omega^2(\omega^2-\omega_c^2)-2\omega^2\omega_p^2+\omega_p^4}{\omega^2-\omega_c^2-\omega_p^2}\frac{1}{c^2} \to 0$ we get the following eigen frequencies

$$\omega^2(\omega^2 - \omega_c^2) - 2\omega^2\omega_p^2 + \omega_p^4 = 0 \ \to \ \begin{cases} \omega_n^2 = \frac{\omega_h^2}{2}\left\{1 + \sqrt{1 - 4(\frac{\omega_p}{\omega_h})^4}\right\}, & \text{for high frequency TM} \\ \omega_n^2 = \frac{\omega_h^2}{2}\left\{1 - \sqrt{1 - 4(\frac{\omega_p}{\omega_h})^4}\right\}, & \text{for low frequency TM} \end{cases} \tag{28}$$

such that $\omega_h^2 = \omega_c^2 + 2\omega_p^2$. It does worth to mention here that the roots in Eq. 28 which define the low and high frequency of the band gap are in fact the poles of α_{11} and α_{12}.

2.1 Low frequency TM-band

For the low frequency TM band when $k \to \infty$ we have $\omega_n = \sqrt{\omega_c^2 + \omega_p^2}$, $\epsilon_{11} = 0$ and $\alpha_{11} = 0$. Therefore we get

$$\lim_{k\to\infty}(A_{n,\phi=0}k) = Re\left\{\frac{i\beta_{12}}{\beta_{11}}\right\}_{\omega_n=\sqrt{\omega_c^2+\omega_p^2}} = -\frac{sgn(\omega_c)}{\sqrt{1+(\frac{\omega_p}{\omega_c})^2}} \tag{29}$$

For the case of $k \to 0$, we have $\omega_n^2 = \frac{\omega_h^2}{2}\left\{1 - \sqrt{1 - 4(\frac{\omega_p}{\omega_h})^4}\right\}$ which is the pole of α_{11} and α_{12} so $\alpha_{11} \to \infty$, $\alpha_{12} \to \infty$, then for $\lim_{k\to 0}(A_{n,\phi=0}k)$ we get

$$\lim_{k\to 0}(A_{n,\phi=0}k) = \lim_{k\to 0}\frac{Re(\frac{i}{(\epsilon_0 c)^2}\{2\frac{\alpha_{12}}{\alpha_{11}}\beta_{11} + (1 + \frac{|\alpha_{12}|^2}{\alpha_{11}^2})\beta_{12}\})}{\frac{1}{(\epsilon_0 c)^2}\{(1 + \frac{|\alpha_{12}|^2}{\alpha_{11}^2})\beta_{11} - 2\frac{\alpha_{12}}{\alpha_{11}}\beta_{12}\} + \frac{\mu}{\alpha_{11}}} \tag{30}$$

in the above equation as $\alpha_{11} \to \infty,\ \alpha_{12} \to \infty$ then $\frac{\alpha_{12}}{\alpha_{11}} = \frac{i\omega_c\omega_p^2/\omega}{\omega^2-\omega_c^2-\omega_p^2}$ and $\frac{|\alpha_{12}|^2}{\alpha_{11}^2} = \frac{i\omega_c^2\omega_p^4/\omega^2}{(\omega^2-\omega_c^2-\omega_p^2)^2}$ and $\frac{\mu}{\alpha_{11}} \to 0$. Therefore we have

$$\lim_{k\to 0}(A_{n,\phi=0}k) = \left\{\frac{Re(\frac{i}{(\epsilon_0 c)^2}\{2\frac{\alpha_{12}}{\alpha_{11}}\beta_{11} + (1 + \frac{|\alpha_{12}|^2}{\alpha_{11}^2})\beta_{12}\})}{\frac{1}{(\epsilon_0 c)^2}\{(1 + \frac{|\alpha_{12}|^2}{\alpha_{11}^2})\beta_{11} - 2\frac{\alpha_{12}}{\alpha_{11}}\beta_{12}\}}\right\}_{\omega_n^2=\frac{\omega_h^2}{2}\left\{1-\sqrt{1-4(\frac{\omega_p}{\omega_h})^4}\right\}} = 1 \tag{31}$$

Therefore the Chern number of the low frequency band is

$$C_n = -\frac{sgn(\omega_c)}{\sqrt{1+(\frac{\omega_p}{\omega_c})^2}} - 1 \tag{32}$$

2.2 High frequency TM-band

For the high frequency band when $k \to \infty$ we get $\omega_n \to \infty$, $\epsilon_{11} = 1$, $\epsilon_{12} = 0$, $\alpha_{11} = 1$, $\alpha_{12} = 0$ and $\beta_{12} = 0$, so $\lim_{k\to\infty}(A_{n,\phi=0}k) = 0$ and for the case of $k \to 0$ we have $\omega_n^2 = \frac{\omega_h^2}{2}\left\{1 + \sqrt{1 - 4(\frac{\omega_p}{\omega_h})^4}\right\}$ again this is a pole of α_{11} and α_{12} so $\alpha_{11} \to \infty,\ \alpha_{12} \to \infty$ and we have

$$\lim_{k\to 0}(A_{n,\phi=0}k) = \left\{\frac{Re(\frac{i}{(\epsilon_0 c)^2}\{2\frac{\alpha_{12}}{\alpha_{11}}\beta_{11} + (1 + \frac{|\alpha_{12}|^2}{\alpha_{11}^2})\beta_{12}\})}{\frac{1}{(\epsilon_0 c)^2}\{(1 + \frac{|\alpha_{12}|^2}{\alpha_{11}^2})\beta_{11} - 2\frac{\alpha_{12}}{\alpha_{11}}\beta_{12}\}}\right\}_{\omega_n^2 = \frac{\omega_h^2}{2}\left\{1+\sqrt{1-4(\frac{\omega_p}{\omega_h})^4}\right\}} = -1 \tag{33}$$

Finally for the high frequency Chern number we have

$$C_n = 0 - (-1) = 1 \tag{34}$$

As it can be seen from Eq. 32, generally the Chern number of the low frequency TM band is non-integer but that of high frequency band is integer.

2.3 TE-Mode

Using same procedure (the details of computation are omitted for conciseness) it is straightforward to show that for TE-Mode we have

$$\Delta C_n = \lim_{k\to\infty}(A_{n,\phi=0}k) - \lim_{k\to 0^+}(A_{n,\phi=0}k) = 0 \tag{35}$$

2.4 Wave vector cutoff for magneto-optic material response

The emergence of a wave vector cutoff is well understood in some materials. For example, a lossless electron gas described by a drift-diffusion model has a spatially dispersive response such that the permittivity seen by the transverse waves is $\epsilon_T/\epsilon_0 = 1 - \omega_p^2/\omega^2$, whereas the permittivity seen by the longitudinal waves is $\epsilon_L/\epsilon_0 = 1 - \omega_p^2/(\omega^2 - \nu^2 k^2)$, where ω_p is the plasma frequency and ν is a parameter with unities of velocity that depends on the diffusion coefficient [4]. Hence, in the limit $k \to \infty$ the longitudinal permittivity approaches the response of the vacuum, i.e., the response to longitudinal waves has a wave vector cutoff. In this specific physical system, the wave vector cut-off for longitudinal oscillations is a consequence of the diffusion effects which act to avoid the localization of the electrons over distances smaller than some characteristic diffusion length. Inspired by this result, we may introduce a high-frequency spatial cutoff by transforming a local material response as

$$\boldsymbol{M}_{reg}(\omega, \boldsymbol{k}) = \boldsymbol{M}_\infty + \frac{1}{1 + k^2/k_{max}^2}\{\boldsymbol{M}(\omega) - \boldsymbol{M}_\infty\} \tag{36}$$

where $\boldsymbol{M}_\infty = \lim_{\omega\to\infty} \boldsymbol{M}(\omega)$. Based on this type of material response, the permittivity tensor components become

$$\epsilon_{11}(k) = \epsilon_{22}(k) = 1 - \gamma\frac{\omega_p^2}{\omega^2 - \omega_c^2},\quad \epsilon_{12}(k) = -\epsilon_{21}(k) = -i\gamma\frac{\omega_c\omega_p^2}{\omega(\omega^2 - \omega_c^2)} \tag{37}$$

such that $\gamma = \frac{1}{1+k^2/k_{max}^2}$.

For this case, in dispersion equation $k^2 = k^2 = \frac{\epsilon_{11}(k)^2+\epsilon_{12}(k)^2}{\epsilon_{11}(k)}(\frac{\omega}{c})^2$, we have $k \to \infty$ if $\epsilon_{11}(k) = 0$ or $\omega_n \to \infty$, So the eigen frequency of the higher TM band is $\omega_n \to \infty$ and that of lower frequency band comes from the zero of $\epsilon_{11}(k)$.

$$\epsilon_{11}(k) = 1 - \gamma\frac{\omega_p^2}{\omega^2 - \omega_c^2} = 0 \to \omega_n = \sqrt{\omega_c^2 + \gamma\omega_p^2} \tag{38}$$

when $k \to \infty$ then $\gamma \to 0$ so for low frequency band eigen frequency we get $\omega_n = \lim_{\gamma\to 0}\sqrt{\omega_c^2 + \gamma\omega_p^2} = |\omega_c|$.

For the case of $k \to 0$ ($\gamma \to 1$) we have same dispersion equation when we had no wave vector cut-off and the eigen frequencies are going to be those in Eq. 4. Therefore nothing changes in this case.

As it was mentioned, For low frequency TM band as $k \to \infty$ ($\gamma \to 0$) we have $\omega_n = |\omega_c|$ so $\epsilon_{11}(k) = 0$, $\alpha_{11}(k) = 0$ and we get

$$\lim_{k\to\infty}(A_{n,\phi=0}k) = Re\left\{\frac{i\beta_{12}(k)}{\beta_{11}(k)}\right\}_{\omega_n=|\omega_c|} \tag{39}$$

such that $\beta_{11}(k) = 1+\gamma\omega_p^2\frac{\omega^2+\omega_c^2}{(\omega^2-\omega_c^2)^2}$, $\beta_{12} = 2i\gamma\omega_c\omega_p^2\frac{\omega}{(\omega^2-\omega_c^2)^2}$ so the contribution form $k \to \infty$ in low frequency TM band is

$$\lim_{k\to\infty,\ \gamma\to 0}(A_{n,\phi=0}k) = Re\left\{\frac{i\beta_{12}(k)}{\beta_{11}(k)}\right\}_{\omega_n=|\omega_c|} = \lim_{\gamma\to 0}\frac{-2\gamma\omega_c^2\omega_p^2}{1+2\gamma\omega_c^2\omega_p^2} = 0 \tag{40}$$

For this band if $k \to 0$ ($\gamma \to 1$) then wave vector cut-off does nothing and we have

$$\lim_{k\to 0}(A_{n,\phi=0}k) = \left\{\frac{Re(\frac{i}{(\epsilon_0 c)^2}\{2\frac{\alpha_{12}}{\alpha_{11}}\beta_{11}+(1+\frac{|\alpha_{12}|^2}{\alpha_{11}^2})\beta_{12}\})}{\frac{1}{(\epsilon_0 c)^2}\{(1+\frac{|\alpha_{12}|^2}{\alpha_{11}^2})\beta_{11}-2\frac{\alpha_{12}}{\alpha_{11}}\beta_{12}\}}\right\}_{\omega_n^2=\frac{\omega_h^2}{2}\left\{1-\sqrt{1-4(\frac{\omega_p}{\omega_h})^4}\right\}} = 1 \tag{41}$$

so for low frequency band we get following Chern number

$$C_n = 0 - 1 = -1 \tag{42}$$

For the high frequency TM band as $k \to \infty$ ($\gamma \to 0$), as it was explained before, we get $\lim_{k\to\infty}(A_{n,\phi=0}k) = 0$ and when $k \to 0$ ($\gamma \to 1$) again we have

$$\lim_{k\to 0}(A_{n,\phi=0}k) = \left\{\frac{Re(\frac{i}{(\epsilon_0 c)^2}\{2\frac{\alpha_{12}}{\alpha_{11}}\beta_{11}+(1+\frac{|\alpha_{12}|^2}{\alpha_{11}^2})\beta_{12}\})}{\frac{1}{(\epsilon_0 c)^2}\{(1+\frac{|\alpha_{12}|^2}{\alpha_{11}^2})\beta_{11}-2\frac{\alpha_{12}}{\alpha_{11}}\beta_{12}\}}\right\}_{\omega_n^2=\frac{\omega_h^2}{2}\left\{1+\sqrt{1-4(\frac{\omega_p}{\omega_h})^4}\right\}} = -1 \tag{43}$$

so for high frequency band we get following Chern number

$$C_n = 0 - (-1) = +1 \tag{44}$$

3 Supplementary Videos

Video S1: Clockwise SPP rotation at the interface of positively biased (along $+z$) plasma covered by silver. The color bar represents the strength of the electric field.

Video S2: Counterclockwise SPP rotation at the interface of negatively biased (along -z) plasma covered by silver. The color bar represents the strength of the electric field.

References

[1] Mário G. Silveirinha and Stanislav I. Maslovski, *Exchange of momentum between moving matter induced by the zero-point fluctuations of the electromagnetic field*, Phys. Rev. A 86, 042118; DOI: 10.1103/PhysRevA.86.042118 (2012).

[2] S. Raghu and F. D. M. Haldane, *Analogs of quantum-Hall-effect edge states in photonic crystals*, Phys. Rev. A 78, 033834; DOI: 10.1103/PhysRevA.78.033834 (2008).

[3] Mário G. Silveirinha, *Chern invariants for continuous media*, Phys. Rev. B 92, 125153; DOI: 10.1103/PhysRevB.92.125153 (2015).

[4] G. W. Hanson, E. Forati, and M. G. Silveirinha, *Modeling of spatially-dispersive wire media: Transport representation, comparison with natural materials, and additional boundary conditions*, IEEE Trans. Antennas Propag. 60, 4219-4232 (2012).
