# Supplementary figures and images for: The effects of three-dimensional defects on one-way surface plasmon propagation for photonic topological insulators comprised of continuum media
Source: Sci Rep. 2016 Jul 21;6:30055. doi: 10.1038/srep30055 (PMC4956765; doi:10.1038/srep30055)

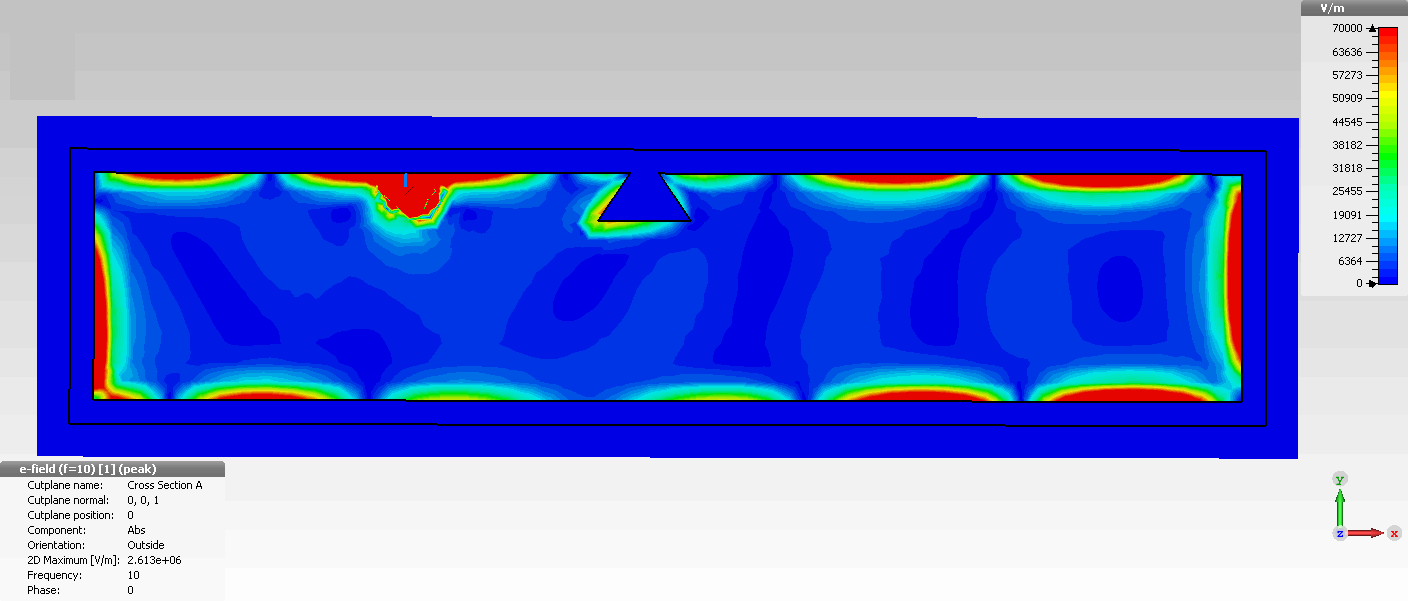

Supplement: Supplementary Video S1 [file srep30055-s2.gif]

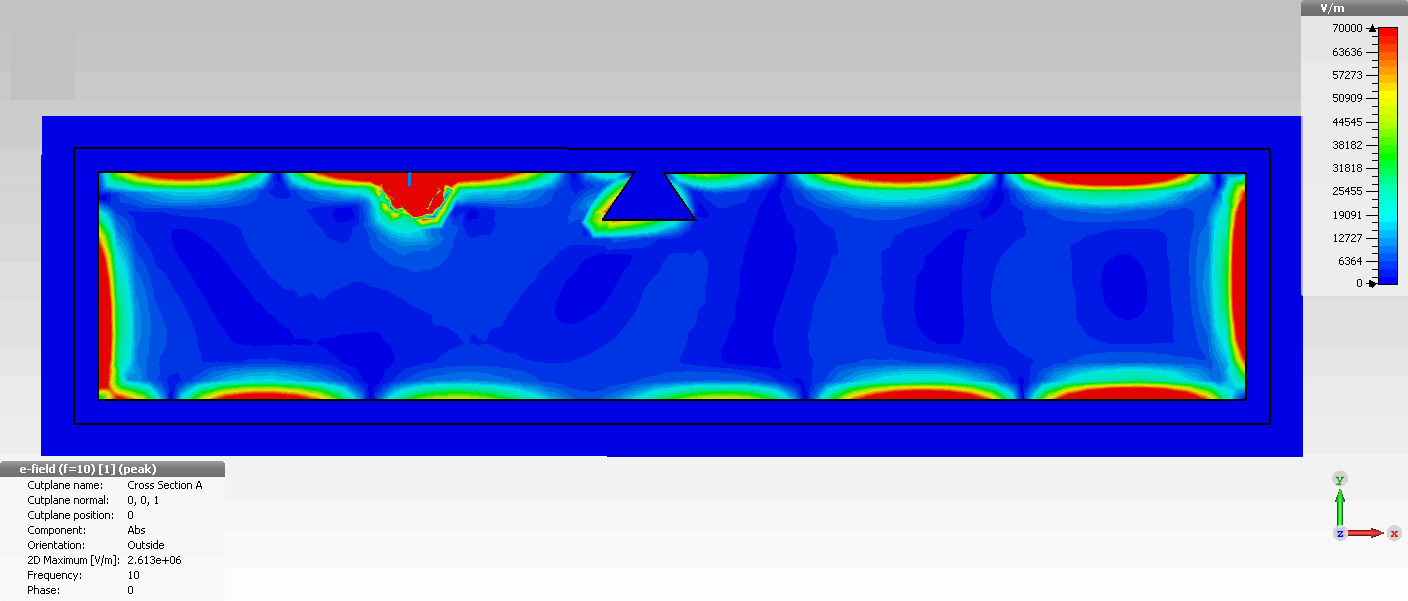

Supplement: Supplementary Video S2 [file srep30055-s3.gif]
